# A qualitative study of Swedish fathers’ experiences of becoming a father during the COVID-19 pandemic

**DOI:** 10.18332/ejm/146082

**Published:** 2022-03-25

**Authors:** Michael B. Wells, Joline Svahn, Karolina Svedlind, Ewa Andersson

**Affiliations:** 1 Department of Women’s and Children’s Health, Karolinska Institutet, Stockholm, Sweden

**Keywords:** COVID-19, pregnancy, perinatal care, prenatal care, participation, expectant fathers

## Abstract

**INTRODUCTION:**

Expectant fathers want to participate in perinatal care. COVID-19 policies restrict their access to care, but it is unknown how these policies have affected them. The aim of this study is to explore the perinatal care given to and wanted by expectant and new fathers during the COVID-19 pandemic in Sweden.

**METHODS:**

The current study used an inductive qualitative design where 14 expectant or new fathers participated in a video- or telephone-based semi-structured interview. Interviews lasted 20 minutes, on average. The collected data were analyzed using content analysis.

**RESULTS:**

Two main themes were reported: 1) ‘Being left out, but trying to remain positive’, and 2) immediate consequences related to restrictions. Expectant fathers were not able to attend as many perinatal visits as they wanted to, due to the COVID-19 restrictions on non-birthing parents. Expectant fathers regretted and felt discouraged that they could not support their partner during visits and not follow their baby’s growth and development. Furthermore, they faced uncertainties and stress regarding whether or not they could attend the birth of their child. Fathers reported how their exclusion negatively impacted the entire family.

**CONCLUSIONS:**

Expectant and new fathers felt that their level of participation in prenatal care was negatively impacted by the Swedish policies imposed on them during the COVID-19 pandemic. Fathers were physically and emotionally excluded, resulting in receiving little direct care support, and lacked companionship with other parents. Fathers provided suggestions and alternatives on how to increase their participation with individual midwives and from an organizational level.

## INTRODUCTION

The COVID-19 pandemic has had profound and unprecedented impacts on healthcare systems, including within perinatal care^1,2^. The adverse effects of COVID-19 within the perinatal field include rising rates of maternal deaths, stillbirth, and mental ill-health^3^. Involving fathers in perinatal care can be an effective and low-cost way to reduce anxiety and promote maternal–fetal attachment in expectant mothers^4^. However, understanding how COVID-19 has affected expectant and new fathers is much less studied.

Before the pandemic, many expectant and new fathers felt excluded and marginalized from perinatal care^5-7^. Across many countries, COVID-19 policies have now restricted fathers’ involvement in perinatal care by physically excluding them from participating; leaving fathers feeling unwanted, unhelpful, and isolated at a time when emotional familial connectedness is needed^8,9^. Since fathers’ presence and involvement in perinatal care positively benefits mothers, infants, and themselves^6,10^, excluding fathers from participating may have consequences beyond the father himself.

Transiting into parenthood is a tumultuous and life-affirming transition for expectant and new parents, but is often mitigated by various professional and social supports^5,7,11^. However, these supports may be limited due to health professionals not having any formalized care guidelines for fathers^11^. Professional support to fathers has now further been reduced due to the exclusionary COVID-19 policies that ban fathers from attending prenatal, birth and postnatal care^12^. Excluding fathers has resulted in receiving conflicting information about their partners’ and baby’s health and a reduction in the quality of care provided, resulting in a sense of isolation, psychological distress, and reduced bonding with their baby^13,14^.

### Swedish COVID-19 perinatal policies

Perinatal care guidelines in Sweden changed as COVID-19 spread through the population. Local regions adapted new COVID-19 policies based on the infection rate within their own population and, therefore, policies across different regions of the country varied and changed over time, up to the point where it was unclear what the current guidelines were and what rules applied^15^. While fathers typically attend prenatal, labor/birth, and postnatal clinics, as well as prenatal parent education classes with their expectant partner, under new COVID-19 policies, expectant mothers were not allowed to have anyone else present during these visits and the parental education classes were often cancelled. However, since the World Health Organization promotes birthing women to have a support person present, the labor/birth policies were the first to shift; however, immediately following the birth, the support person, often the father, had to leave the hospital, thus unable to spend the first night together as a family.

## METHODS

The current study used a qualitative inductive study design with semi-structured interviews to help fulfil the aim.

### Participants

Convenience sampling methods were used to recruit participants. Participants were recruited by an information letter on the ‘Män för Jämställdiget’ (Men for Gender Equality) website, which contained information about the study and the researcher’s contact information. Participants were also recruited from two (JS and KS) of the authors’ personal Facebook pages, where a poster with information about the study and contact information for the authors was posted. This poster was shared by Facebook friends. In both recruitment methods, the expectant or new fathers would reach out to the researchers to express their interest in participating.

Inclusion criteria were: 1) living in Sweden, 2) being able to speak Swedish enough to participate in an interview, 3) aged >18 years, and 4) currently being in the third trimester of pregnancy or having had a newborn infant on or after March 2020 (i.e. the baby had to have been born during the COVID-19 pandemic). All potential participants who contacted the researchers met the inclusion criteria, and all but one father participated in the study. The one father who dropped out did so because he had a baby when the interview was to take place. Participants were sent an information letter about the study via email, which they were asked to read through before the scheduled interview.

### Data collection

The data were collected through individual semi-structured interviews during February 2021. All authors designed and constructed the interview guide, while three researchers (JS, KS and EA) completed the interviews. All interviewers followed the interview guide and conducted the interviews through a recorded video link or telephone. The interviewers did not know any of the participants prior to interviewing them.

A pilot interview was conducted to test the interview guide and was not included in the analysis. A total of fourteen interviews were conducted. During the interviews, the interviewer and participant were the only ones present on video link/telephone, except in two cases, where two fathers had their child present. Participants were informed and consented to their interview being recorded at the start of the interview. Fathers always participated in the study from their home.

### Interview guide

The semi-structured interview guide asked participants about their experiences of expecting a baby and participation in prenatal care during the COVID-19 pandemic. If something needed to be clarified, supplementary questions were asked, where the authors tried to encourage the participants to talk further about the topic. Hypothetical saturation was reached by the thirteenth interview, with an additional interview occurring to further ensure saturation. The interviews ranged from 12 to 37 minutes, with the average interview taking 20 minutes. All interviews were transcribed verbatim.

### Data analysis

The data were analyzed by three authors using content analysis, employing an inductive approach as described by Elo and Kyngäs^16^. According to content analysis, the analysis consists of three main phases: preparation, organizing and resulting phase. The preparation phase started to create a Word document and two of the authors read the material several times to make sense of the whole. In the organizing phase, open coding was conducted by writing comments in the margins, creating a coding sheet, grouping the codings, and organizing categorization and abstraction. Preliminary themes were created and all authors discussed the findings. Final themes and categories were further constructed after more iterations of codings and group discussions. All authors agreed on the final analysis.

## RESULTS

Participants consisted of seven expectant fathers and seven fathers of newborns. The participants’ ages ranged 26–47 years, with a median age of 34 years. The current pregnancy for the participants was either their first (primiparous, n=7) or second (multiparous, n=7). All participants had completed upper secondary school and ten of them also completed higher education. All participants lived together with the mother. Four of the fathers were born outside Sweden and had lived in Sweden between 12 and 24 years.

The results of the content analysis consisted of two themes including: 1) ‘Being left out, but trying to remain positive’, and 2) immediate personal and familial consequences related to restrictions (Table 1).

**Table 1:** Main themes and generic categories

*Main themes*	*Generic categories*
‘Being left out, but trying to remain positive’	Quality of care has decreased Midwives are gatekeepers Problem solving to be more involved
Immediate personal and familial consequences related to restrictions	Emotions of being left outside Strengthened coparenting Unable to bond with baby Unprepared and worried

### ‘Being left out, but trying to remain positive’

Within this theme, fathers discussed how their care had changed before and during COVID-19 period. Due to new regulations, participants discussed how midwives adapted to include them, as well as stated their suggested father-inclusive adaptations that adhere to COVID-19 social distancing policies and in their families’ best interest.

*Quality of care has decreased*

Fathers experienced a lack of opportunity to be a support person to their partner. Multiparous fathers discussed how they were more active in care and could discuss the care they received, as well as their care needs more easily with their partner during their first pregnancy than during their current pregnancy. By being able to be present during prenatal visits gave fathers the opportunity to feel like this was a joint effort, rather than under the current restrictions, where fathers noted that their partner was left alone with her experiences and needing to take full responsibility:

*‘... that maybe I get sick and cannot be part of the birth and that would be a great thing, it would be very sad of course to miss it … then above all that, my wife cannot get the support from me because … it is difficult to replace me with another person, because we know each other without and within […] and this [pregnancy] is a very big tough thing that you go through as a woman also of course and then you want your support and security.’* (P2)

Multiparous fathers stated that they had a high level of participation in prenatal care with their first child and perceived their participation as exceptionally positive. They emphasized how their previous experience helped them cope with not being allowed to attend prenatal visits during their current pregnancy. In fact, they sympathized with primiparous fathers who were not allowed to participate in the same manner. Some multiparous fathers reflected on what primiparous fathers were missing:

*‘I feel very sorry for fathers who are expecting their first child. That they do not get the opportunity that I had with my first child, which I now miss with my second child.’* (P10)

Fathers described the need to talk with other parents and to share their own experiences of pregnancy. Multiparous fathers who had previously received parental education felt that it was a pity that primiparous fathers did not have access to preparation, such as being able to test breathing techniques and how to make the pregnancy and birth easier for their partner.

Multiparous fathers further reported that they appreciated all of the information they learned from these classes during their first pregnancy and relied heavily on that knowledge to get them through this second pregnancy. They further appreciated having contact with other parents during pregnancy. The desire to meet other parents was something that all fathers described, and they wanted to exchange thoughts about pregnancy, especially as childbirth approached. Primiparous fathers described that they would have appreciated parental groups to support them and to meet other parents. Not allowing fathers to attend parental education put them in precarious situation of lacking knowledge about pregnancy, birth, and becoming a father, as illustrated by one father:

*‘... there are no parent groups or anything, then I have had to take to the internet, I have joined one of the few father groups on Facebook. There are not that many actually. [...] no parent groups, no one to exchange ideas with, share, or meet.’* (P14)

*Midwives are gatekeepers*

Fathers believed that midwives held different attitudes concerning whether or not to include fathers in the prenatal period. They reported having positive experiences of the midwife adapting to the restrictions and still managing to include fathers:

*‘She [the midwife] used to ask how it was with me, so she asked my partner, how is it with me and how am I, and so on. [...] Even though I was never present, it was still as if she still included me in the pregnancy, even though I was not present.’* (P7)

However, other fathers described midwives as inflexible and denying fathers opportunities to maintain feelings of participation. Since they were not allowed to attend, some fathers experienced insufficient communication with midwives:

*‘... I have not actually spoken a single word with, with… a doctor or midwife or nurse during or throughout this pregnancy.’* (P12)

*Problem solving to be more involved*

Fathers had different opinions about how the prenatal clinics handled their care during the COVID-19 pandemic. Fathers sometimes reported that they felt confident that healthcare organizations were doing everything they could to adapt to the situation. At the same time, fathers also felt that prenatal care was unequally delivered depending on whether the facility was a state or private clinic. Fathers suggested that organizational matters were a question of management, and not solely dependent on the midwife.

For example, some fathers said that they chose private clinics just so they could participate in prenatal care:

*‘We actually applied privately to participate in two ultrasounds [...] and it was probably our little one ... yes, trying to do something, something good from a bad situation as well.’* (P12)

Fathers expressed that they wanted to be involved in some way and described different solutions to be more included in prenatal care. For example, they suggested that: 1) midwives write a short summary after every visit, 2) that the midwife allow the mother to record the fetal heart rate, and 3) that the management provide the midwife with a webcam to enable web-based video meetings:

*‘You should think a little extra. We have friends who are in the same period as us, and they go to a private clinic. There, the partner can follow, there are other conditions they have. Contrary to what the state offers, of course it is state and private, but should it really be this injustice and different?’* (P10)

One father who wanted to have a video call during the appointment noted that the midwife found this unacceptable:

*‘We suggested using it [video call] ourselves, but it was rejected [by the midwife]. They did not want to [have a video call] because they claimed they could not concentrate.’* (P14)

Fathers believed that finding solutions was important and vital to their involvement in following their child’s growth and development, as well as to hear how their pregnant partner was doing. However, fathers noted that these strategies automatically put additional expectations on the expectant mother. They experienced that she felt alone with her experiences of pregnancy, and that she had a huge responsibility to inform her partner about what happened at the visit.

### Immediate personal and familial consequences related to restrictions

Fathers reflected on the immediate consequences of not being allowed to attend prenatal visits. They also described what they missed when they were not able to participate in prenatal visits and parental classes.

*Emotions of being left outside*

One immediate consequence of the restrictions was that fathers were not allowed to attend prenatal visits, which directly impacted their emotional well-being. Fathers’ emotional experiences of prenatal care included feelings of being left out and fear of not being able to participate during labor. Fathers expressed that the restrictions at the prenatal clinics affected their level of participation. They stated that the restrictions directly forced them not to participate or be involved in prenatal care:

*‘As it is now, it feels very much … like I am completely … completely eh, outside … Pregnancy in a slightly more old-fashioned traditional way or what to say.’* (P1)

The feelings of being left out of prenatal care services was a central focus by the fathers. Fathers who felt left out found it difficult to be a part of the pregnancy and to understand what was happening, both with their partner and with their child. This led to fathers sometimes describing feeling distant from their partner.

Fathers described their desire to participate in prenatal care, as well as how Swedish family policies are aimed around gender equality. Fathers mentioned that they felt societal pressure to be involved, while at the same time, being denied access to that involvement. One primiparous father had an expectation that he might be able to participate, but gradually understood that this was not possible:

*‘... I wanted to be involved [...]. Society is built on the intention that you should have two parents and if you have it, that both should be involved. There are many things that have changed historically to give the father more participation in parenting and it is not really reflected in this year, I can say.’* (P 4)

*Strengthened coparenting*

Fathers reported that because they could not physically attend prenatal visits, they needed to better communicate with their partner regarding how their baby was growing and developing.

Fathers therefore reported spending more time discussing the outcomes of the visits with their partner, as well as preparing questions that concerned them, so that their partner could ask those questions during her next prenatal visit. This constant planning for visits and paraphrasing the outcomes of visits, helped strengthen the couple’s relationship:

*‘We have become welded together [...] our little team has had to be strengthened.’* (P5)

*Unable to bond with baby*

Fathers reported that hearing and seeing their infant was important to them for the bonding process, especially since they could not feel the baby growing inside their bodies, like the expectant mother:

*‘So it was something I had been looking forward to, to hear my daughter's heartbeat […] I think you might not really get as emotionally involved because of it [not being able to attend and participate].’* (P7)

Fathers had expectations that were not being fulfilled and this affected their possibility to get involved in the pregnancy. One multiparous father expressed that he felt less bonded with his baby:

*‘... I feel much more distanced from the growing fetus that is in the stomach now, in every way…’* (P 5)

*Unprepared and worried*

One consequence of the restrictions was not being able to attend prenatal education classes. Fathers described that they lacked information on how to prepare for labor and how to take care of the baby. In the maternity ward, midwives assumed that parents had participated in parent groups, but this had not been offered. The parents did not really know much about a birth. Fathers stated that more responsibility was placed on mothers to help to prepare for the upcoming birth, which could have led to a more stressful situation:

*‘And it was also almost as if the midwives were used to people having such an education … There they just assumed that we had taken one of the courses, but we did not. We did not really know much about the birth.’* (P4)

Concerns about becoming sick before the labor were recurrent in the fathers’ stories. If they had COVID-19 symptoms, they would not be able to participate in the birth of their child, which made fathers feel especially anxious:

*‘It's a worry about not being allowed to participate, but I have tried to process it then and tried to find solutions and not make too much of it. But of course, it's a big, big thing.’* (P2)

## DISCUSSION

During the 2020 COVID-19 pandemic, expectant fathers in Sweden experienced a low level of participation in perinatal care due to the restrictions put in place. Fathers named several negative consequences of these policies, including negative impacts to their own health, the mother’s mental health, their ability to bond with their baby, and the reductive societal message that fathers are a distant secondary parent to the mother. When midwives, especially those at private prenatal clinics, made efforts to include fathers, fathers reported these experiences as extremely positive for the whole family and greatly appreciated their efforts. Fathers tried to problem solve their involvement in prenatal care, while still adhering to social distancing guidelines.

Even though Sweden is one of the most gender equal countries in the world^17^ and politically discusses the importance of gender equality, including in parenting^18^, the pandemic has undermined some of this progress^15^. Fathers in the current study reported this juxtaposition, which left them feeling like unimportant secondary parents. The lack of national routines and guidelines for how to support expectant and new fathers was glaringly missing, resulting in large variations of care. As a result, fathers were often left to the whims of the individual midwife, who was the gatekeeper of their involvement. For example, the current data show that some midwives were flexible and adapted care policies, such as having video or telephone conferencing, recording the infant’s heartbeat, printing notes on the baby’s growth and development, and answering the father’s questions through the expectant mother, while other midwives were firm in not allowing these activities to happen. As such, some fathers noted that they switched prenatal clinics, especially to privately run clinics, to receive more inclusive care. These findings however are not central to Sweden, as other pandemic-related research on fathers from the US^19,20^ and Israel^21^ also emphasize a greater need for providing family-centered prenatal care, to help minimize long-term consequences of non-involvement^8^. Future research is needed to better understand the consequences and long-term outcomes of the exclusionary policies pertaining to non-birthing parents’ involvement, including fathers and LGBTQ parents, in perinatal care.

In researching expectant fathers in the US, Poulos et al.^20^ found that their stress primarily developed from COVID-19 related issues, such as disease risk, safety protocols, and long-term health effects. The present Swedish study noted that expectant fathers were worried about contracting COVID-19 symptoms during the birth of their baby, as all fathers wanted to attend this event. However, fathers were also stressed regarding not being able to attend prenatal visits. In line with the current results, previous research shows that exclusionary restrictions of care negatively affected both expectant and new mothers and fathers, throughout the perinatal period, by forcing the expectant mother to go through perinatal care by herself, creating loneliness and sadness for both parents, as well as highlighting that the non-birthing parent is not important enough to receive professional support^15^. Since there is an association between received perinatal professional support and the father’s depression symptoms^22^. Since men’s suicide rates may increase when they are less connected to their family^23^, more efforts should be made to include, support and promote fatherhood throughout the perinatal period. While more research exists on fathers, other non-birthing parents, such as LGBTQ parents, also require perinatal professional support but were also neglected of this support due to COVID-19 restrictions in Sweden. Further research is therefore important to understand the similarities and differences in support needs of fathers and LGBTQ non-birthing parents, especially due to COVID-19 restrictions.

Previous research states that hearing the fetal heart beat and being present during the ultrasound are important prenatal bonding experiences for expectant fathers who do not have the chance to consistently feel their baby’s growth and movement, like expectant mothers^5,24^. The current study highlighted that because fathers were excluded from perinatal care, they did not have these opportunities to bond with their infant and felt less connected to them. However, literature reviews show that fathers bond best when attending the birth and participating in postnatal care^25^. Postnatal father–infant bonding is associated with improved breastfeeding rates, reduced cognitive delays, and promoting weight gain in preterm infants^26,27^. However, during the COVID-19 pandemic, fathers could perceive a dysfunctional interaction with their infant, resulting in added stress^21^. Future policies should be put in place to ensure that the whole family unit can remain together postnatally, as doing so benefits all members of the family.

Having a strong social network is important for parents’ emotional and mental stability^28^. Similar to the current studies’ findings, research from the US stipulated that fathers felt socially isolated by not being able to meet other expectant parents and share experiences^20^. While Sweden has a strong prenatal parent education program, where parents can meet and socialize^28^, they were often discontinued during COVID-19. These policies can place expectant fathers at risk for social isolation, and consequently place more vulnerability on fathers. Further efforts could be made to try to include and support fathers’ social support network by offering, for example, digital-based parent groups or offering telephone-based peer support programs, which have been shown to be beneficial amongst mothers^29^.

### Limitations

The current study is one of the first to assess fathers’ perceptions of prenatal care during COVID-19 and is the first in Sweden. However, the study is relatively small and qualitative studies may not generalize to other settings, especially in countries where fathers were not excluded from attending prenatal and postnatal care. Additionally, participants were recruited from a gender-equity Facebook page (Men for Gender Equality), and therefore these fathers may be more involved in perinatal care. The study’s participants though consist of a wide age range, first- and multi-time fathers, and fathers with different-origin backgrounds, which can increase transferability. Another potential limitation is that the interviews were relatively short in length. However, they contained content-rich data, resulting in two themes. In addition, a semi-structured interview guide was used and was pilot tested; thus, ensuring that each participant discussed similar topics, adding to the study’s credibility. Regarding researcher reflexivity, two authors held preconceived notions that fathers should be included in prenatal care, as had been standard practice prior to the COVID-19 pandemic, while two other authors held more neutral opinions on fathers’ involvement. Nevertheless, the authors all agreed on the overall themes and categories. In fact, dependability was enhanced, as three authors conducted the interviews and all authors helped code, categorize, and analyze the data. The authors had different experience levels (ranging from novice to experts), lending robustness to the findings.

## CONCLUSIONS

Expectant and new fathers who all had pregnancies during the 2020 COVID-19 pandemic reported that it was difficult for them to receive any type of perinatal care, including prenatal parent education groups. This lack of care resulted in perceived negative outcomes for the expectant father and mother, including deteriorating mental health, lack of father–infant bonding, and more pressure on expectant mothers to be the primary parent. While fathers suggested several solutions that still adhered to social distancing guidelines, such as having midwives write notes about their baby’s growth and development, allowing the expectant mother to record the visits, especially their infants’ heartbeat, and using webcams, they perceived midwives to be the gatekeepers of care, where care differed based on the type of organization (public vs private) and on the individual midwives’ perceptions of fathers. National guidelines regarding how perinatal care is delivered to expectant and new fathers might help control for variability and further encourage expectant father involvement.

## Data Availability

The data supporting this research are available from the authors on reasonable request.

## References

[1] Thapa SB, Mainali A, Schwank SE, Acharya G (2020). Maternal mental health in the time of the COVID-19 pandemic. Acta Obstet Gynecol Scand.

[2] Suwalska J, Napierała M, Bogdański P (2021). Perinatal Mental Health during COVID-19 Pandemic: An Integrative Review and Implications for Clinical Practice. J Clin Med.

[3] Chmielewska B, Barratt I, Townsend R (2021). Effects of the COVID-19 pandemic on maternal and perinatal outcomes: a systematic review and meta-analysis. Lancet Glob Health.

[4] Mokaberian M, Dehghanpouri H (2021). THE EFFECT OF FATHERS' PARTICIPATION IN PRENATAL CARE ON ANXIETY AND MATERNAL-FETAL ATTACHMENT IN UNWANTED FIRST PREGNANT WOMEN DURING COVID-19 PANDEMIC. Article in Persian. Nursing And Midwifery Journal.

[5] Wells MB (2016). Literature review shows that fathers are still not receiving the support they want and need from Swedish child health professionals. Acta Paediatr.

[6] Xue WL, Shorey S, Wang W, He HG (2018). Fathers' involvement during pregnancy and childbirth: An integrative literature review. Midwifery.

[7] Johansson M, Fenwick J, Premberg A (2015). A meta-synthesis of fathers' experiences of their partner's labour and the birth of their baby. Midwifery.

[8] Lista G, Bresesti I (2020). Fatherhood during the COVID-19 pandemic: an unexpected turnaround. Early Hum Dev.

[9] Bradfield Z, Wynter K, Hauck Y (2021). Experiences of receiving and providing maternity care during the COVID-19 pandemic in Australia: A five-cohort cross-sectional comparison. PLoS One.

[10] Plantin L, Olukoya AA, Ny P (2011). Positive Health Outcomes of Fathers' Involvment in Pregnancy and Childbirth Paternal Support: A Scope Study Literature Review. Fathering.

[11] van Vulpen M, Heideveld-Gerritsen M, van Dillen J, Oude Maatman S, Ockhuijsen H, van den Hoogen A (2021). First-time fathers' experiences and needs during childbirth: A systematic review. Midwifery.

[12] Diamond RM, Brown KS, Miranda J (2020). Impact of COVID-19 on the Perinatal Period Through a Biopsychosocial Systemic Framework. Contemp Fam Ther.

[13] Vasilevski V, Sweet L, Bradfield Z (2021). Receiving maternity care during the COVID-19 pandemic: Experiences of women's partners and support persons. Women Birth.

[14] Nespoli A, Ornaghi S, Borrelli S, Vergani P, Fumagalli S (2021). Lived experiences of the partners of COVID-19 positive childbearing women: A qualitative study. Women Birth.

[15] (2021). Att föda barn under en pandemi - Vittnesmål. Report in Swedish.

[16] Elo S, Kyngäs H (2008). The qualitative content analysis process. J Adv Nurs.

[17] (2020). Gender Equality Index 2020.

[18] Ellingsæter AL, Leira A (2006). Politicising parenthood in Scandinavia: Gender relations in welfare states.

[19] Recto P, Lesser J (2021). Young Hispanic fathers during COVID-19: Balancing parenthood, finding strength, and maintaining hope. Public Health Nurs.

[20] Poulos NS, García MH, Bouchacourt L, Mackert M, Mandell DJ (2021). Fatherhood during COVID-19: fathers' perspectives on pregnancy and prenatal care. J Mens Health.

[21] Ben-Yaakov O, Ben-Ari OT (2021). COVID-19-Related anxieties and parenting stress among first-time mothers and fathers in their first year of parenthood. Psychol Health.

[22] Wells MB, Aronson O (2021). Paternal postnatal depression and received midwife, child health nurse, and maternal support: A cross-sectional analysis of primiparous and multiparous fathers. J Affect Disord.

[23] Dehara M, Wells MB, Sjöqvist H, Kosidou K, Dalman C, Sörberg Wallin A (2021). Parenthood is associated with lower suicide risk: a register-based cohort study of 1.5 million Swedes. Acta Psychiatr Scand.

[24] Walsh TB, Tolman RM, Neal Davis R, Palladino CL, Romero VC, Singh V (2014). Moving Up the "Magic Moment": Fathers' Experience of Prenatal Ultrasound. Fathering.

[25] Scism AR, Cobb RL (2017). Integrative Review of Factors and Interventions That Influence Early Father-Infant Bonding. J Obstet Gynecol Neonatal Nurs.

[26] Bronte-Tinkew J, Carrano J, Horowitz A, Kinukawa A (2008). Involvement Among Resident Fathers and Links to Infant Cognitive Outcomes. Journal of Family Issues.

[27] Garfield CF, Isacco A (2006). Fathers and the well-child visit. Pediatrics.

[28] Forslund Frykedal K, Berlin A, Barimani M (2021). Föräldragrupper inom mödra- och barnhälsovård: forskning, tillämpning och metoder om ledarskap för välfungerande grupper. Article in Swedish.

[29] Dennis CL (2013). Peer support for postpartum depression: volunteers' perceptions, recruitment strategies and training from a randomized controlled trial. Health Promot Int.

